# Alternative Non-Destructive Approach for Estimating Morphometric Measurements of Chicken Eggs from Tomographic Images with Computer Vision

**DOI:** 10.3390/foods13244039

**Published:** 2024-12-14

**Authors:** Jean Pierre Brik López Vargas, Katariny Lima de Abreu, Davi Duarte de Paula, Denis Henrique Pinheiro Salvadeo, Lilian Francisco Arantes de Souza, Carlos Bôa-Viagem Rabello

**Affiliations:** 1 Institute of Geosciences and Exact Sciences, São Paulo State University (UNESP), Rio Claro 13506-900, SP, Brazil; jean.vargas@unesp.br (J.P.B.L.V.); davi.duarte@unesp.br (D.D.d.P.); 2 Zootechnics Department, Federal Rural University of Pernambuco (UFRPE), Recife 52171-900, PE, Brazil; katariny.lima@ufrpe.br (K.L.d.A.); carlos.rabello@ufrpe.br (C.B.-V.R.)

**Keywords:** morphometric data extraction, eggs quality, poultry, computer tomographic images, 3D image segmentation, deep learning

## Abstract

The egg has natural barriers that prevent microbiological contamination and promote food safety. The use of non-destructive methods to obtain morphometric measurements of chicken eggs has the potential to replace traditional invasive techniques, offering greater efficiency and accuracy. This paper aims to demonstrate that estimates derived from non-invasive approaches, such as 3D computed tomography (CT) image analysis, can be comparable to conventional destructive methods. To achieve this goal, two widely recognized deep learning architectures, U-Net 3D and Fully Convolutional Networks (FCN) 3D, were modeled to segment and analyze 3D CT images of chicken eggs. A dataset of real CT images was created and labeled, allowing the extraction of important morphometric measurements, including height, width, shell thickness, and volume. The models achieved an accuracy of up to 98.69%, demonstrating their effectiveness compared to results from manual measurements. These findings highlight the potential of CT image analysis, combined with deep learning, as a non-invasive alternative in industrial and research settings. This approach not only minimizes the need for invasive procedures but also offers a scalable and reliable method for egg quality assessment.

## 1. Introduction

The eggshell is crucial for food safety due to its ability to withstand physical and pathogenic challenges from the external environment and broken or cracked eggs are identified as one of the main causes of losses, as bacteria can penetrate the shell [1]. Eggshell quality has been proven to be useful in developing strategies that reduce the risk of foodborne pathogens, since eggs with more resistant shells offer greater protection against pathogen penetration and contamination of their internal contents [2]. Studies show that the quality of the cuticle and the thickness of the shell influence the antibacterial efficiency of eggs. The authors observed that a thicker cuticle layer and a greater shell thickness protected bacterial penetration and that antibacterial efficiency can reach up to 98% when the cuticle opacity exceeds 27.5% and the shell thickness exceeds 340 μm [3].

Furthermore, the quality of the eggshell is essential for egg production chain [1], since it needs to be strong enough to avoid breakage during packaging, transportation, and handling, since eggshell breakage can represent between 8% and 10% of total production, leading to significant economic losses [4]. The efficiency of this physical barrier depends on the structural integrity of the eggshell, making the quality a determining factor in the production, marketing and consumption of eggs. These properties result from the complex structure of the shell, which is highly organized due to the controlled interactions between the mineral and organic matrix [4] and any defect in the eggshell structure can result in losses in its quality [1]. Internal or external factors such as genetics, age, oviposition time, housing systems and nutrition can affect the quality of the eggshell [4].

Among the characteristics related to eggshell quality, thickness is a measure of shell resistance. This parameter is particularly relevant and is evaluated with the aim of reducing egg breakage [5,6], contributing to the protection of the egg against contamination. The length and width of the egg are also important to estimate its shape, which can directly impact the distribution of force on the eggshell. Eggs with more balanced length-width ratios tend to be more resistant to breakage, which is crucial for production, transportation and food safety [7]. In addition, egg shape can influence consumer acceptance, as more elongated or flattened eggs can be perceived as having lower aesthetic quality. Therefore, morphometric variables can be used to evaluate eggshell quality. These variables determine characteristics such as shape index, volume, and surface area of the egg.

Destructive techniques are also used to evaluate the efficiency of its physical barrier, including resistance to eggshell breakage and thickness [7,8,9]. However, these techniques are applied individually to each egg, require trained personnel, and involve partial or total destruction of the eggs during analysis. Therefore, the search for alternatives to evaluate morphometric variables and eggshell quality is important, as new assessment methodologies can allow for more detailed and accurate assessments of eggshell properties, enabling the identification of factors that affect eggshell quality more effectively. In the context of large-scale production, these strategies become more relevant, as small gains in accuracy can result in significant reductions in losses due to broken or damaged eggs, which negatively impact the production chain, both in terms of consumer safety and economic losses.

In this context, the use of computed tomography (CT) combined with computer vision techniques can represent an effective method for analyzing eggshell quality. Studies have used CT to measure the total volume of the egg and shell, correlating these variables with others obtained through destructive methods. Significant results were found in the mechanical strength of the shell, volume, weight, height, and width of the egg, concluding that the experimental model is effective in evaluating factors that influence egg quality [10]. Thus, the use of CT appears to be a promising approach to assess egg quality. The main contribution of this work is to propose an innovative method to obtain morphometric measurements of chicken eggs in a non-invasive way, through the analysis of computed tomography images using computer vision techniques with a deep learning approach, and the creation of a CT Image Dataset.

The paper is organized as follows: A description of the manual and destructive techniques used to measure chicken egg features, a description of the CT image acquisition process and the labeling procedure, the data preprocessing, the deep learning models approach, and the hyperparameters used are shown in Section 2. Details of the results of the experiments using the modified deep learning models to perform the image segmentation and measurement estimation tasks are shown in Section 3. An in-depth discussion of the destructive measurement methods used in comparison with the non-invasive ones used in this paper is shown in Section 4, and the results of Section 3 are discussed. Finally, in Section 5, some concluding remarks are presented.

## 2. Materials and Methods

This section will describe some of the classic measurement techniques for chicken egg characteristics as well as the procedure for acquiring CT images and labeling them, in addition to the deep learning models chosen for this study and the steps followed for their respective training and the equipment that was used for this process.

### 2.1. Classical Techniques

**Figure 1:**
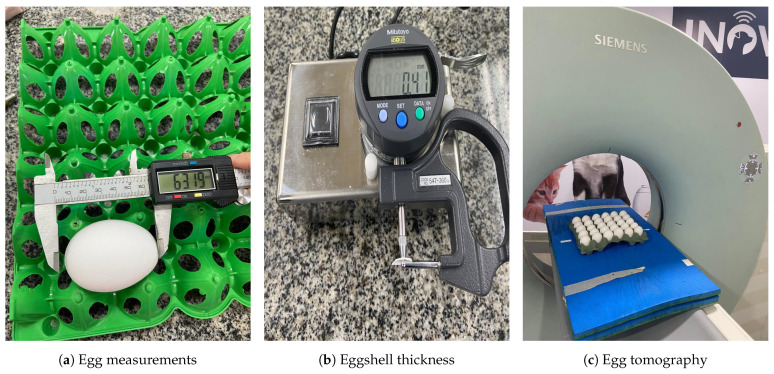
Measuring Equipment: (**a**) Egg measurements, length and width measured with a digital caliper (**b**) Eggshell thickness, measured with digital micrometer (**c**) Acquisition of images of chicken eggs by computed tomography.

In order to assess egg quality variables, eggs from light laying hens of the Dekalb White strain, reared at the Poultry Research Laboratory of the Federal Rural University of Pernambuco, will be used. These eggs come from a research project approved by the UFRPE Animal Ethics Committee (CEUA 6000110221). The evaluations were conducted on fresh eggs and eggs stored at room temperature for 7, 14, 21, and 28 days, with 30 eggs assessed at each period, totaling 150 eggs. To generate the data required to train and validate the computer vision models, the eggs were analyzed for length (mm) and width (mm) as described in Figure 1a, and shell thickness (mm) as described in Figure 1b.

Egg length and width measurements were taken using a digital caliper (684132, LeeTools, Recife, Brazil)). To determine the thickness of the shell, it was washed and then dried at room temperature for 48 h.The measurement was then determined by averaging the thickness of the basal, equatorial and apical regions using a digital micrometer (547-360, Mitutoyo, Kyoto, Japão). These equipment are shown in Figure 1.

The egg volume (*V*) was estimated using the equation proposed by [7], $V=\left(0.6057-0.0018\cdot W\right)\cdot L\cdot W^{2}$ where *W* and *L* represent the width and length of the egg, respectively.

### 2.2. Image Acquisition Procedure and Labeling

The tomographic analyzes of the eggs were performed using a helical CT scanner (Siemens Spirit, Siemens Medical Solutions, Malvern, USA), calibrated prior to the examinations, as shown in Figure 1c. The images were acquired through transverse slices with a thickness of 3 mm, a slice interval of 2 mm, a pitch of 1.6, 130 kV, 30 mA, and reconstruction filters for soft tissues.The images were obtained using the smallest possible slice thickness (3 mm) and a slice interval of 2 mm, accordance with the technical limitations of the equipment used.

Two image acquisition sessions were conducted: the first included trays containing fresh eggs and eggs stored for 7 days, while the second included trays with eggs stored for 14, 21, and 28 days. All trays contained 30 eggs each.

**Figure 2:**
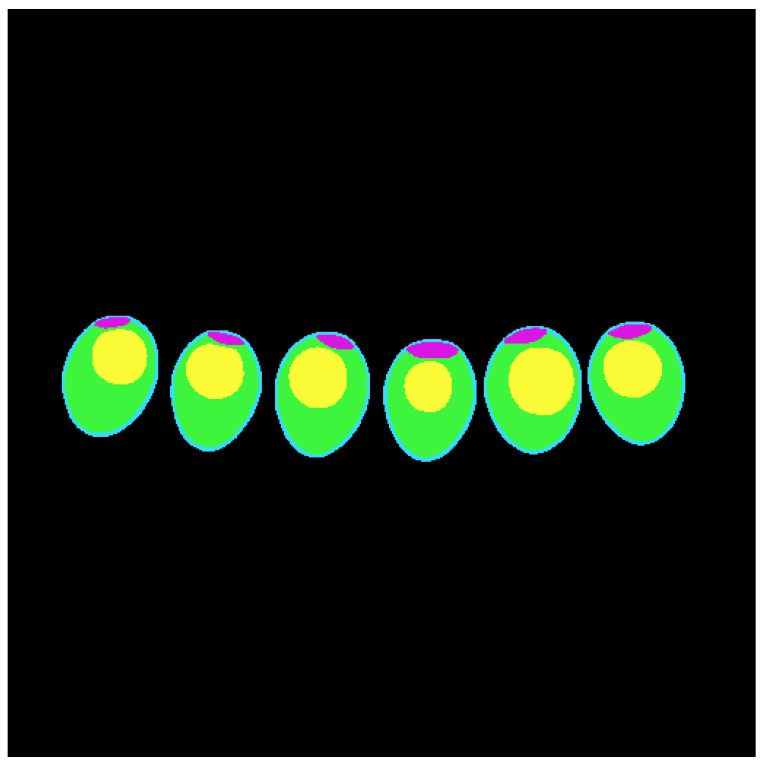
Labeling of egg structures made in CVAT.

An average of 12 to 15 images per row of eggs were used, as the slices from the rows are utilized in the Computed Tomography (CT) process. Each tray contained 5 rows of 6 eggs. The slices obtained from the CT images underwent a annotation process using the Computer Vision Annotation Tool (CVAT). During this step, the regions of interest were manually identified and labeled, encompassing the shell, air cell, albumen, and yolk. Each of these structures was precisely delineated, as illustrated in Figure 2. The colors assigned to each region were as follows: green for the albumen, yellow for the yolk, purple for the air cell, and a distinct shade of blue for the eggshell. Approximately 585 images were used for this annotation step.

### 2.3. Data and Preprocessing

From the image acquisition procedure in the previous Section 2.2, we obtain 89 slices of 512 × 512 voxels for each egg tray due to the chosen depth of the voxels of 3 mm, which means that there are about 11 slices per row of the tray, corresponding to a 3D Image of an egg. The images are in IMA (Siemens DICOM files) format, a format similar to DICOM (Digital Imaging and Communications in Medicine).

Due to their large size, the images had to go through several preprocessing stages to reach the 40 (slices) × 140 (height) × 90 (width) measurements for each individual egg. First, the egg images were separated into rows. The second step to reach 40 slices per row was to add background images in similar amounts, both in front and behind the original images. Finally, the images were cropped to 140 × 90 in the appropriate position for correct viewing. After a prior analysis it was found that with a minimum of 40 slices, the models worked correctly.

The volume measurement of a voxel in the image was obtained by calculating the side of the voxel by dividing the size 333 mm by the number of pixels 512, resulting in 0.650390625 mm. Therefore the volume of each voxel is 0.650390625 mm × 0.650390625 mm × 3 mm = 1.269023798 mm^3^.

Since the images are from tomography, they contain values outside the RGB range of a conventional image, between 0 and 1600, for which they were normalized to have values between 0 and 255. This helped improve the results obtained. From the 150, were chosen, separated and normalized 122 3D images, considering each one contains 40 depth slices, they were separated into 3 sets (training, validation and test) in percentages of (80/10/10)% [11], to use them in the training processes of the models.

### 2.4. Overview of Models Architecture

The 3D U-Net proposed in [12] is composed of an encoder-decoder architecture, with a contraction part (encoder) and an expansion part (decoder), using 3D convolutional layers in both parts. The encoder progressively extracts high-level features while reducing input volume, consists of 4 stages, each with two 3 × 3 × 3 convolutional layers followed by ReLU activation and 2 × 2 × 2 max pooling. Each stage reduces the spatial dimensions by half while doubling the number of feature channels.

The decoder has 4 stages, each with a 2 × 2 × 2 upsampling operation followed by two 3 × 3 × 3 convolutional layers and ReLU activation, these are correspondingly concatenated with the features sampled from the different stages of the encoder with the so-called skip connections. This is done to preserve the spatial information. The encoder-decoder combination architecture takes the shape of the letter U, and from there it earned its name.

The FCN architectures called FCN-16, which generally uses a backbone such as VGG or Resnet. As the U-NET, it has two parts. In the downsampling part it has seven blocks. Block 1 and 2 contain two 3D convolutional layers with their Relu activation function and at the end a 3D maxpooling layer. Blocks 3, 4 and 5 have an additional convolutional layer than the previous blocks. Blocks 6 and 7 only contain a 3D convolutional layer with its Relu activation function and a 3D dropout layer. In the upsampling part, it has two 3D convolutional layers followed by two transposed 3D convolutional layers concatenated with the skip connections from the downsampling part.

### 2.5. Models Training and Hyperparameters Tunning

Model training experiments were performed on a desktop computer with Intel Core i9-12900F processor, 64 GB RAM, 4 TB storage and NVIDIA GeForce RTX 4090 graphics card with 24 GB of RAM.

The U-Net 3D [13] and FCN 3D [14] models were chosen because they provide good results in the area of segmentation of real and synthetic images [15,16] and are not too large in relation to the number of parameters. They were used to receive the 3D images and estimate the 3D segmentation masks so that from there the morphometric measurements of class volume, height, width and shell thickness can be estimated by applying the voxel counting method per class. The second method used was to modify the aforementioned networks, adding an additional head parallel to the final part of the up-sampling, in order to directly estimate measurements of (height, length and shell thickness).

After obtaining the segmentation results, we initially considered training the networks from scratch for both tasks(segmentation and measurements estimation) considering a weighting between them, but the result was not satisfactory. That is why we decided to train the models in stages, the first stage for segmentation, freezing the values of the model weights, and in the second stage only training the part of the estimation of the morphometric measurements.

**Figure 3:**
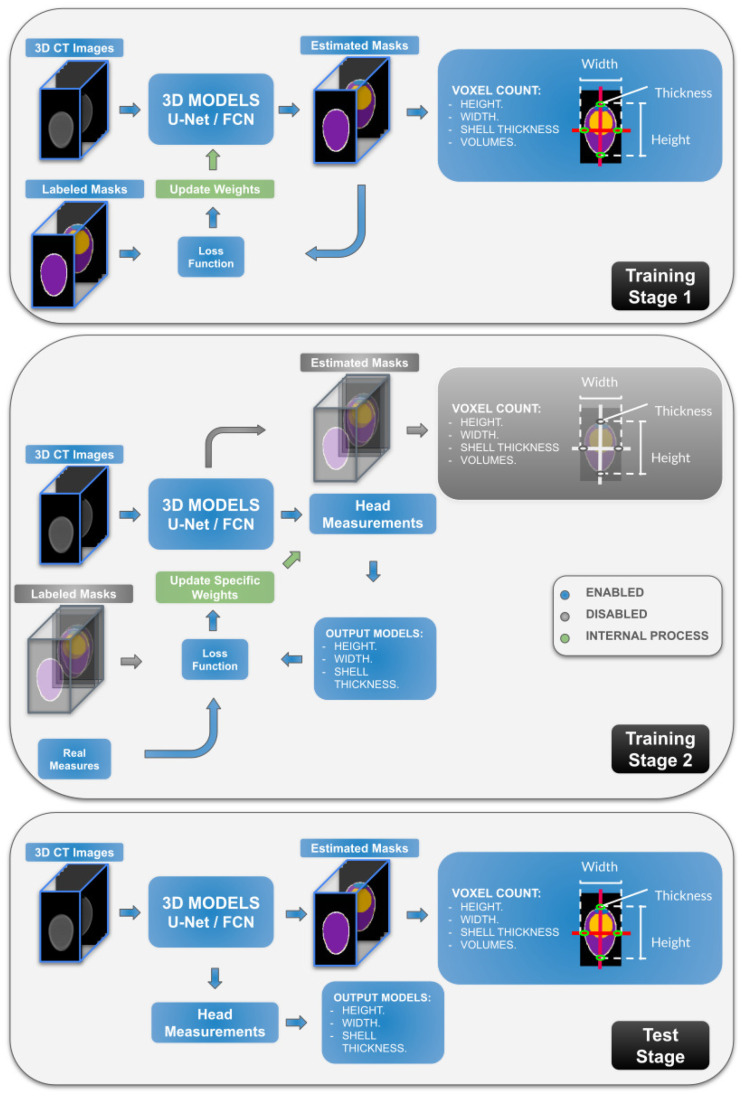
Pipeline of Estimated Morphometrics Measurements since the Inputs (Images) to Outputs (Estimated Masks and Morphometrics Measures) of the Models.

Figure 3 shows the pipeline sequence of the data from the input of the models with the preprocessed images and the output of the models showing the estimated segmented masks and the direct estimation of morphometric measurements.

In Algorithm 1, the training procedure is depicted. It expects a $model_{\theta }$, e.g., the U-NET 3D or the FCN 3D, with θ representing the trainable parameters. Training the model is equivalent to modifying the parameters θ so that the model can output good results in production. The algorithm requires the $model_{\theta }$ initialized with random values of θ, but in line 10, it returns the model with trained values for the set θ. The algorithm also expects a dataset $D=\{s_{1},s_{2},\ldots ,s_{n}\}$, where *n* is the number of samples and $s_{i}=\left\{e_{i},m_{i}\right\}$ represents an individual sample, with $e_{i}$ being the 3D CT egg image and $m_{i}$ the 3D segmentation mask. In line 1, the epoch looping is presented, with *E* denoting the total training epochs. In line 2, batches of *B* random samples are acquired from the dataset D. In this first training stage, all the model parameters are trainable, and the task used is semantic segmentation. In this way, lines 3 and 4 retrieve the 3D CT egg image and the 3D segmentation mask from the batch. In line 5, the model makes a prediction of a 3D segmentation given the 3D CT egg image. In line 6, this prediction is compared with the actual 3D segmentation mask (label), returning a loss measure. In line 7, the parameters θ of the $model_{\theta }$ are updated to improve future predictions.
**Algorithm 1** Training Stage 1**Require:** 
D, $model_{\theta }$                   ▹ Dataset  1:**for** epoch **in** {0,1,⋯,E} **do**  2:    **for all** batch **in** D **do**  3:        eggs←batch[0]              ▹ 3D CT egg image  4:        seg_mask←batch[1]       ▹ 3D CT Segmentation Mask  5:        $predicted\_mask\leftarrow model_{\theta }\left(eggs\right)$      ▹ Makes a prediction  6:        loss←loss(seg_masks,predicted_mask)  7:        **model**.backprop(loss)  8:    **end for**  9:**end for**10:**Output** $model_{\theta }$

The Algorithm 2 depicts the training stage 2. It requires a dataset $D^{\prime }=\left\{s_{1}^{\prime },s_{2}^{\prime },\ldots ,s_{n}^{\prime }\right\}$, where *n* is the number of samples, and $s_{i}^{\prime }=\left\{e_{i},o_{i}\right\}$, with $e_{i}$ being the 3D CT egg image and $o_{i}$ the morphological measures. In line 1, the entire model is set to be non-trainable, i.e., its layers are set to not require gradients (require_grad(False)). In line 2, new layers are appended to the end of the model to output the desired morphological measures. These layers are called the head, and they are configured to be trainable (attach_head(require_grad=True)). In this step, the only trainable layers are this morphological head; all the other layers are kept frozen. Lines 3 to 9 are similar to Algorithm 1, except for lines 6 and 7, because now the label and the predicted value are the morphological measures, not the 3D segmentation mask.
**Algorithm 2** Training Stage 2**Require:** 
D′, $model_{\theta }$                     ▹  Dataset  1:$model_{\theta }$.require_grad(False)  2:$model_{\theta }$.attach_head(require_grad=True)  3:**for** epoch **in** {0,1,⋯,E} **do**  4:    **for all** batch **in** D **do**  5:        eggs←batch[0]                ▹ 3D CT egg image  6:        measures←batch[1]           ▹ Morphological measures  7:        $predicted\_measures\leftarrow model_{\theta }\left(eggs\right)$       ▹ Makes a prediction  8:        loss←loss(measures,predicted_measures)  9:        **model**.backprop(loss)10:    **end for**11:**end for**12:**Output** $model_{\theta }$

Table 1 shows the hyper-parameters obtained after performing several tests. These hyper-parameters are those with which the model performs well and produces better results. The selection of these hyperparameters was chosen in different intervals, for example for the “learning rate” the chosen range was between 0.01 and 0.00001, in the case of the epochs it was chosen between 100 and 2000, the “batch size” between 10 and 25, depending on the memory capacity, in our case 25 for the U-Net model and 15 for the FCN model. The impact on the selection of these hyperparameters influences the obtaining of the minimum cost function, for example a high “learning rate” can reach a minimum cost function value faster but for that same reason it can skip some values, without reaching the global minimum.

### 2.6. Morphometric Measurements

**Figure 4:**
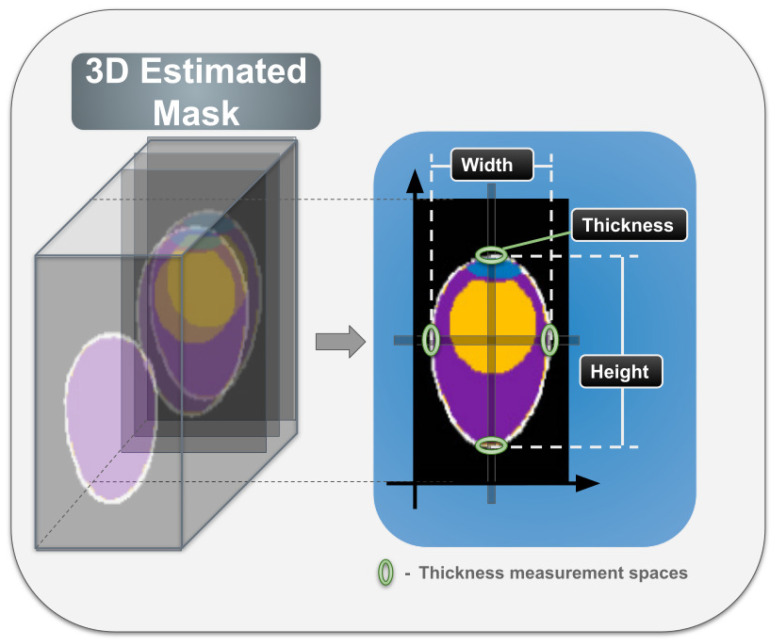
Approach to counting the number of voxels for estimating morphometric measurements.

The first approach considers the number of voxels estimated for each class shown in Figure 4. For all cases, an intermediate slice of the 3D egg image is selected. In the case of height, a point is fixed on the horizontal axis and the voxels that do not correspond to the background are measured. In the case of length, the same procedure is carried out by fixing a point midway on the vertical axis. To measure shell thickness, the same points are considered on both the vertical and horizontal axes and an average is taken of the 4 points that intersect with the estimated voxels of the shell class. These first measurements are compared with the measurements obtained in the same way from the labeled masks. The second approach is to calculate these morphometric measurements directly from the model output by adding a head at the end of the upsampling of the models, so that the same models are responsible for estimating the measurements.

## 3. Results

The results presented in this section provide a comprehensive evaluation of the proposed method, focusing on the segmentation accuracy and morphometric measurements obtained from 3D computed tomographic images. Through experimentation and analysis, we evaluate the performance of our deep learning models in both qualitative and quantitative terms.

**Figure 5:**
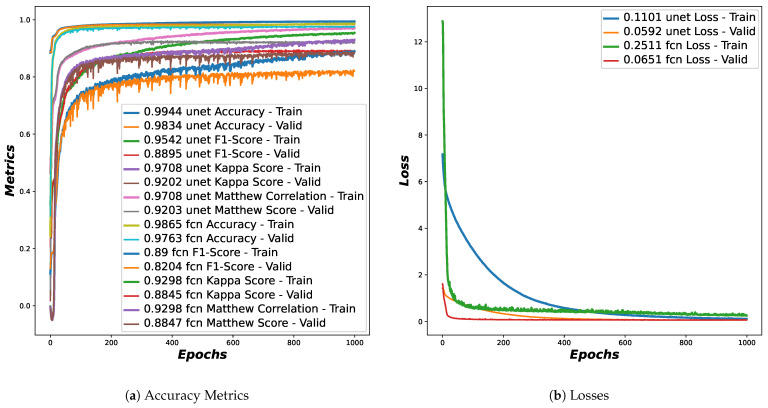
Metrics of the segmentation stage: (**a**) Accuracy metrics of the training and validation sets of the U-Net and FCN models. (**b**) Loss metrics of the training and validation sets of the U-Net and FCN models.

Figure 5a,b show the behavior of the accuracy metrics and loss functions across epochs for the training and validation sets of 3D CT images, as a result of the training stage of the U-Net 3D and FCN 3D algorithms. Within the accuracy metrics we consider the variations of F1-Score, Matthew Correlation, Kappa Score and Accuracy. It is observed that these metrics are mostly close to 1, thus showing adequate performance. In the case of the loss function, it is observed that it reaches values close to 0, also showing that the models are learning adequately. In training models, these graphs are essential and widely used because they are a guide to know if the model is learning the task at hand or if, on the contrary, we need to vary some hyperparameters to improve learning. It also helps us to know which training epoch number is the best to stop. It is important to highlight that the models were trained end-to-end.

The values of all accuracy metrics achieved in the last epoch with the fully trained U-Net and FCN networks are shown in Table 2.

**Figure 6:**
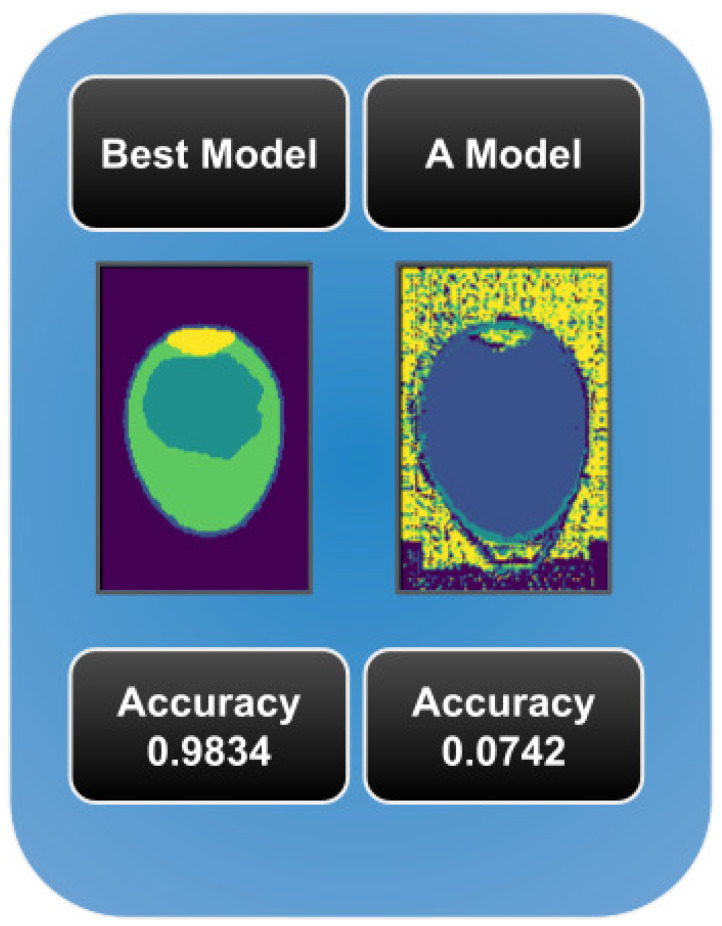
Comparison between a slice (**right**) with low accuracy and a slice (**left**) with high accuracy.

Figure 6 shows a comparison between slices with results from different metrics, it can be seen that low accuracy means wrong segmentation.

Table 3 shows the measurements obtained by the approach of counting the number of voxels for each class from the labeled masks (ground truth). It also shows the number of estimated voxels, thus calculating the total volume per class, as the sum of the voxels volumes for each class. These values correspond to the total of the test set, considering that percentage values greater than 100% in the fourth column indicate that more voxels corresponding to that class were detected.

**Figure 7:**
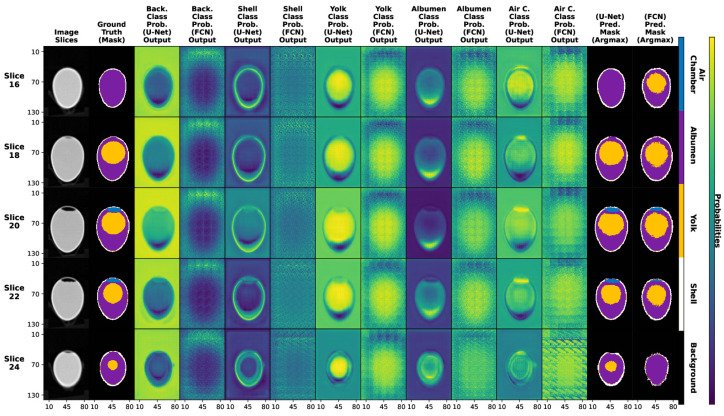
Comparison of real tomographic image slices (column 1), corresponding labeled mask slices (column 2), probability outputs of the networks for each class (column 3–12), showing in yellow the most probable class and the final predictions for the U-Net and FCN networks (column 13–14).

Figure 7 shows an overview of the comparison between the slices of a 3D sample of the test set, the resulting slices of the network as probabilities for each class and, the last two columns, the result of the extraction of the *argmax* function with which we obtain the class with the highest probability.

Table 4 and Table 5 shows the average morphometric measurements of height, width and shell thickness obtained by two approaches. Considering the average of the accuracy percentages with respect to the measures estimated directly at the output of the models, we obtained an average estimate of the performance of each model. We can observe that the U-Net model has a better performance by obtaining an average accuracy percentage of 101.65%, compared to the results of 95.39% of the FCN model. The values shown for 11 eggs are the total for the test set which corresponds to 10% of the overall sample database. From these results we can show that, the FCN model demonstrated lower error rates compared to the U-Net model, including MAE (0.50 vs. 2.24), RMSE (0.69 vs. 2.90), and MAPE (8.37% vs. 10.05%), indicating superior accuracy in predicting egg measurements. Furthermore, the *t*-test *p*-values for FCN predictions (*p* > 0.05) suggest no statistically significant differences between FCN predictions and the ground truth values, confirming the reliability of the FCN model. In contrast, U-Net showed statistically significant differences (*p* < 0.05) for most measurements, suggesting that its predictions deviate more from the actual values.

## 4. Discussion

Eggs are considered the cheapest and healthiest source of animal protein in many countries and represented a complete food because they are rich in amino acids, fatty acids, minerals and vitamins. They also contain antioxidant, anti-inflammatory, immunomodulatory and anticancer components [17,18,19,20], and are considered functional foods [21,22] due to their beneficial effects on human health, along with their basic nutritional impact. However, outbreaks of food poisoning are also frequently associated with egg consumption, due to their contamination with pathogenic bacteria such as *Salmonella enteritidis*, *Bacillus cereus* and *Escherichia coli* [23,24].

Despite this, the egg has efficient antibacterial barriers such as the cuticle, eggshell, egg membranes and antimicrobial agents present in the egg white. However, the egg yolk and albumen represent very suitable environments for microbial development [25].

In this context, the eggshell has proteins in its matrix with effective antimicrobial activities against a wide range of bacteria, including *Salmonella enteritidis*, *Escherichia coli*, *Pseudomonas aeruginosa* and *Staphylococcus aureus*. The inner and outer shell membranes, made up predominantly of collagen, play a crucial role in microbiological protection. In particular, the inner membrane has a high concentration of lysozyme, an enzyme with a potent bacteriolytic action, which acts as an additional barrier, making it difficult for microorganisms to penetrate inside the egg [3].

With regard to the eggshell, our study assessed the thickness, width and height of the eggshell. The relationship between shell thickness and microbiological contamination of the egg is still a matter of debate. Although some studies do not relate bacterial contamination to thinner eggshells [26,27], ref. [2] found greater contamination of eggs by *E. coli* in eggs with thinner eggshells and attributed the contradictions between their studies to the thickness of the shells of the eggs evaluated in these studies. According to the authors in the studies that did not find a relationship between shell thickness and microbiological contamination, the eggshells were very thick (greater than 386 μm), making bacterial penetration difficult, and they emphasized that in eggs with thinner shells (less than 340 μm), shell thickness is an important factor that can contribute to egg contamination. Furthermore, the thickness of the shell can also influence the resistance to breaking during transport and handling, preventing secondary contamination that could compromise the quality and safety of the food.

Egg height and width were assessed in our study and are measures used to determine the egg shape index [28]. Round or abnormally long eggs do not fit well in egg boxes and are more prone to breakage than normal-shaped eggs [29].Research indicates that eggshell strength is influenced by several factors, including egg shape, size, and shell thickness [30]. Altuntaş and Şekeroğlu [31] demonstrated that the force required to break the cascade is specifically correlated with the egg shape index, indicating that rounder shapes have greater strength. Additionally, studies have shown that eggs with a higher shape index, i.e., those closer to a spherical shape, have stronger shells compared to eggs with elongated or irregular shapes [32]. These results highlight the importance of morphometric characteristics in assessing the structural quality of the egg, influencing both its durability during transport and its protection against possible contamination.

Several methods are used to measure eggshell quality, the most common being shell thickness, resistance to breakage, electron microscopy and specific gravity. Of these methods, the first three require breaking the egg and separating the contents of the eggshell and only the last does not require breaking the egg, being a method that uses flotation of the eggs in varying densities of salt water, being classified according to the density at which they float [33]. In addition, they require prior training and skilled labor, thus limiting the likelihood of them being easily secured within the industrial chain. In view of this, non-destructive techniques that facilitate quality analysis are essential, as they can provide precision, speed and immediate results. Consequently, several studies have been conducted to explore different non-destructive methods for the rapid and reliable assessment of product quality. In addition, advances in data analysis have made it possible to estimate eggshell quality using non-destructive, rapid and environmentally friendly techniques [34]. In view of this, there is growing interest in automating and simplifying eggshell quality analysis methods, with the aim of optimizing these processes.

Thus, ref. [35] analyzed eggshell characteristics using computer vision and developed an algorithm capable of calculating various quality indicators, such as morphological characteristics (length and width) and the degree of coloration, using imaging and ovoscopy methods. The authors obtained favorable results when comparing the algorithm with the manual analysis method, obtaining a correlation coefficient of 0.93 for the shape index measured by both methods based on morphological measurements and concluded that the automatic analysis of the external characteristics of hen eggs using computer vision allowed the calculation of morphological parameters and the numerical estimation of the structural characteristics of the shell.

In addition, ref. [36] developed an intelligent system that uses a combination of fuzzy logic and computer vision techniques to classify eggs based on parameters such as defects and size. By segmenting images captured by a digital camera with three CCD sensors, 752 × 582 pixel with rate of 30 frames per second and applying image processing algorithms, the authors were able to determine the size of eggs, cracks and fractures in the eggshell, achieving an accuracy rate of 95% for size detection, 94% for crack detection and 98% for fracture detection.

The purpose of our study was to use computerized tomographic images to estimate morphometric measures of hen eggshell quality, contributing to the state of the art in the use of computer vision associated with AI(Artifitial Intelligence). In this sense, we can assume that both models tested (U-Net and FCN) achieved good metrics, despite the short data set, so we can assume that the results will be better by increasing the number of images in the training set, however, it is worth noting that the proposal could be extended to other conditions, being a general methodology. Any validation for these other conditions would be necessary in future studies. We believe that although chicken eggs have shapes that are considered simple, the biggest challenge was working with tomographic images in three-dimensional form. The decision to crop the images was also to save memory when training the models, and to evaluate evaluate a greater number of images at the same time. Regarding the results, we can say that the U-Net model was more effective in terms of performance despite being a smaller model and, as expected, it was more effective in terms of processing speed, quite possibly for this same reason.

The results of the first approach to estimate volume measures for U-Net and FCN (Table 3), show that the total number of voxels is close to the estimated number of voxels per class, with some exceptions. Specifically, the numbers of voxels corresponding to the estimated yolk and eggshell classes show a greater deviation for the case of U-Net estimates, while FCN demonstrated better performance, particularly for the eggshell and yolk classes, as reflected in the percentage values. The recall metric provides a more realistic indication of voxel classification accuracy, showing the voxels correctly classified in each class. The results suggest although while both models are effective, there are still areas for improvement, especially in segmenting of specific regions, such as the air chamber and yolk in both models. In Table 4 and Table 5 we compare two methodologies for estimation physical measurements, one based on voxel counting, and the other based on direct estimation of model outputs. In both methods, the estimates for height, length, and thickness are generally close, with the exception of thickness measurements, where some differences are observed. It’s important to note that the reference thickness used for the voxel counting method comes from labeled images, which may introduce a slight deviation from the actual measurement, especially since this reference relies on an approximation rather than a ground truth.

The practical implications of these results highlight the potential usefulness of 3D tomography in assessing egg quality; by providing accurate volumetric and morphometric measurements, CT imaging can offer a non-invasive and highly accurate alternative to traditional manual techniques. This is particularly useful in scenarios where invasive methods could damage the product or are time-consuming. Accurate estimation of parameters such as shell thickness, air chamber size, and overall egg volume is crucial for quality control in the poultry industry, as these measurements are indicative of egg freshness and integrity. In addition, the ability to automate the process through deep learning models such as U-Net and FCN reduces the need for labor-intensive manual measurements, increasing efficiency and performance. However, further refinements are needed to improve model accuracy, especially for more challenging regions such as the air chamber and yolk, to fully realize the potential of this approach in industrial applications. However, practical applications of this method may face several challenges. Reliance on high-resolution CT imaging equipment incurs substantial costs, making widespread adoption on an industrial scale difficult. Furthermore, the segmentation accuracy of deep learning models can be affected by variations in CT image quality caused by different imaging conditions, egg shapes, or operator settings, requiring robust preprocessing techniques and larger, more diverse datasets for training. Furthermore, although the current study achieved high accuracy, it is important to account for potential errors in identifying specific features due to model biases or limitations in the dataset. Addressing these challenges is essential for the transition from research to practical implementation.

## 5. Conclusions

The use of computed tomography (CT) images presents a promising alternative to conventional techniques for assessing the quality of chicken egg, particularly in obtaining accurate morphometric measurements such as shell thickness, yolk, albumen, air chamber and volume which can contribute to improving food safety for egg consumers. In this study, we applied advanced deep learning models as U-Net and Fully Convolutional Networks (FCN) to analyze 3D tomographic images of eggs, achieving measurement accuracies of up to 98.95%. Compared to traditional destructive methods, CT imaging offers a noninvasive approach that preserves the integrity of eggs while providing reliable and consistent data. Futhermore, the automation of these measurements through deep learning algorithms accelerates the process, making it more efficient and scalable for industrial applications. However, the combination of CT imaging and deep learning provides an effective and non-destructive method for egg quality assessment, with significant potential to improve the manual methods used in both research and commercial settings. Future work could explore improving the segmentation models by incorporating transformer-based architectures and could also try to augment the database using synthetic data to make the system more robust. We could also add images in different conditions such as eggs of different breeds and sizes, as well as add shape constraint measures when performing segmentation, thus reducing errors in pixel identification.

## Figures and Tables

**Table 1:** Accuracy Metric Values on the Training, Validation and Test Sets for the U-Net and FCN Networks.

Hyperparameter	U-Net 3D	FCN 3D
Loss Function	Cross Entropy/MSE *	Cross Entropy/MSE *
Optimizer	Adam	Adam
Learning Rate	0.0001	0.00001
Epochs	1000	1000
Batch Size	25	15
Dropout	0.1	-
Learning Rate (2nd)	0.00001	0.00001
Epochs (2nd)	80	1000

* Mean Square Error (MSE), the second loss function was used in the second stage of model training (Estimation of measurements directly from the output).

**Table 2:** Evaluation Metric Values on the Training, Validation and Test Sets for the U-Net and FCN Networks.

Model	Metrics	Training	Validation	Test
U-Net 3D	Accuracy	0.9944	**0.9834**	**0.9869**
	F1-Score	0.9542	0.8895	0.8483
	Kappa Score	0.9708	0.9202	0.8479
	M. Correlation	0.9708	0.9203	0.8553
	Loss 1	0.1101	0.0592	-
	Loss 2—MSE	3.9900	7.3266	7.9200
FCN 3D	Accuracy	0.9865	0.9763	0.9829
	F1-Score	0.8900	0.8202	0.6346
	Kappa Score	0.9298	0.8845	0.6339
	M. Correlation	0.9298	0.8847	0.6567
	Loss 1	0.2511	0.0651	-
	Loss 2—MSE	2.8979	1.5336	0.4456

The numbers in bold are the best results.

**Table 3:** Volumes of Classes in the Real CT Images of the Test Set.

Model	Class	Total Number of Voxels	Estimated Number of Voxels	% of Mean Estimated Number of Voxels	Mean Precision per Class	Mean Recall per Class	Mean Total Estimated Volume	Mean Total Volume (Equation)
U-Net 3D	Shell	64,293	69,017	(109.65%)	0.7872	0.8524	54.17 (85.27%)	63.51
	Yolk	141,965	141,874	(100.25%)	0.8507	0.8490		
	Albumen	244,697	244,951	(100.30%)	0.8868	0.8885		
	Ar Chamber	14,660	13,695	(96.88%)	0.8934	0.8327		
FCN 3D	Shell	64,293	63,465	(100.65%)	0.7071	0.7044	53.88 (84.84%)	63.51
	Yolk	141,965	141,265	(100.22%)	0.8468	0.8437		
	Albumen	244,697	248,745	(101.88%)	0.8508	0.8648		
	Ar Chamber	14,660	13,595	(88.33%)	0.7516	0.6190		

**Table 4:** Real CT Image Measurements of the Test Set using U-Net.

Model	Samples	Measures	Measurement by Voxels in the Mask	Estimated Measurement by Voxels in the Mask	% of Success per Voxels Measurement	Real Physical Measurement	Estimated Measurement at Output	% of Success at Output Measurement	% of Success at Output per Model
U-Net	Egg 1	Thickness	1.6260	1.4634	(90.00%)	0.3700	0.4479	(121.05%)	107.03%
		Height	59.1855	59.8359	(101.10%)	59.8800	64.5472	(107.79%)	
		Width	45.5273	44.8770	(98.57%)	45.4500	48.7350	(107.23%)	
	Egg 2	Thickness	1.9512	1.7886	(91.67%)	0.3200	0.3876	(118.75%)	
		Height	57.2344	57.8848	(101.14%)	59.5200	61.7045	(103.67%)	
		Width	43.5762	43.5762	(100.00%)	43.4400	46.7352	(107.58%)	
	Egg 3	Thickness	1.3008	1.1382	(87.5%)	0.3900	0.4246	(108.87%)	
		Height	59.8359	59.1855	(98.91%)	59.4200	63.9901	(107.69%)	
		Width	45.5273	45.5273	(100.00%)	43.2800	48.0945	(111.12%)	
	Egg 4	Thickness	1.4634	1.6260	(111.11%)	0.4000	0.4378	(109.45%)	
		Height	58.5352	58.5352	(100.00%)	59.3100	59.5307	(100.37%)	
		Width	42.2754	43.5762	(103.08%)	42.6000	44.5303	(104.53%)	
	Egg 5	Thickness	1.6260	1.3008	(80.00%)	0.3600	0.3516	(97.67%)	
		Height	55.2832	56.5840	(102.35%)	56.8700	61.4720	(98.75%)	
		Width	44.2266	44.2266	(100.00%)	43.9700	46.4149	(97.34%)	
	Egg 6	Thickness	1.3008	1.4634	(100.00%)	0.3700	0.4443	(98.57%)	
		Height	61.7871	61.1367	(100.00%)	61.8000	64.2553	(108.09%)	
		Width	44.2266	44.8770	(98.48%)	44.7700	48.4615	(108.25%)	
	Egg 7	Thickness	1.3008	1.4634	(112.50%)	0.3400	0.3696	(108.71%)	
		Height	57.8848	56.5840	(97.75%)	57.8200	62.4110	(107.94%)	
		Width	44.2266	43.5762	(98.53%)	43.6300	47.3255	(108.47%)	
	Egg 8	Thickness	0.9756	1.3008	(133.33%)	0.2900	0.4598	(158.55%)	
		Height	57.2344	57.8848	(101.14%)	57.5300	62.3857	(108.44%)	
		Width	44.2266	44.8770	(101.47%)	44.2800	47.2127	(106.62%)	
	Egg 9	Thickness	1.1382	1.6260	(142.86%)	0.3600	0.3945	(108.33%)	
		Height	59.1855	58.5352	(98.90%)	59.3300	63.9301	(107.75%)	
		Width	46.1777	46.8281	(101.41%)	46.5800	48.2832	(103.66%)	
	Egg 10	Thickness	1.1382	1.6260	(142.86%)	0.3300	0.2728	(82.67%)	
		Height	56.5840	54.6328	(96.55%)	55.7600	60.0560	(107.70%)	
		Width	42.9258	42.9258	(100.00%)	44.0800	45.7268	(103.74%)	
	Egg 11	Thickness	1.4634	1.3008	(88.89%)	0.3600	0.3209	(89.14%)	
		Height	57.8848	56.5840	(97.75%)	58.2000	61.6495	(105.93%)	
		Width	43.5762	43.5762	(100.00%)	43.5300	46.8414	(107.61%)	
	Mean ± sd	Thickness			(107.34% ± 21.99)			(109.25% ± 19.12)	
		Height			(99.60% ± 1.70)			(105.83% ± 3.24)	
		Width			(100.14% ± 1.35)			(106.01% ± 3.47)	

**Table 5:** Real CT Image Measurements of the Test Set using FCN.

Model	Samples	Measures	Measurement by Voxels in the Mask	Estimated Measurement by Voxels in the Mask	% of Success per Voxels Measurement	Real Physical Measurement	Estimated Measurement at Output	% of Success at Output Measurement	% of Success at Output per Model
FCN	Egg 1	Thickness	1.6260	1.1382	(70.00%)	0.3700	0.3949	(106.72%)	95.39%
		Height	59.1855	59.1855	(100.00%)	59.8800	60.3605	(100.80%)	
		Width	42.9258	44.8770	(104.54%)	45.4500	45.2794	(99.62%)	
	Egg 2	Thickness	1.9512	0.9756	(50.00%)	0.3200	0.3583	(111.97%)	
		Height	57.2344	57.2344	(100.00%)	59.5200	58.9571	(99.05%)	
		Width	43.5762	43.5762	(100.00%)	43.4400	44.1526	(101.64%)	
	Egg 3	Thickness	1.3008	1.3008	(100.00%)	0.3900	0.3054	(78.31%)	
		Height	59.8359	59.8359	(100.00%)	59.4200	59.7758	(100.60%)	
		Width	45.5273	45.5273	(100.00%)	45.5400	44.6681	(98.09%)	
	Egg 4	Thickness	1.4634	1.1382	(77.78%)	0.4000	0.3133	(78.33%)	
		Height	58.5352	57.8847	(98.89%)	59.3100	57.7527	(97.37%)	
		Width	42.2754	42.9258	(101.54%)	42.6000	42.9015	(100.71%)	
	Egg 5	Thickness	1.6260	1.3008	(80.00%)	0.3600	0.2110	(58.61%)	
		Height	55.2832	55.9336	(101.18%)	56.8700	57.4620	(101.04%)	
		Width	44.2266	43.5762	(98.54%)	43.9700	43.0780	(97.97%)	
	Egg 6	Thickness	1.3008	0.9756	(75.00%)	0.3700	0.4490	(121.35%)	
		Height	61.7871	61.1367	(98.95)	61.8000	60.7595	(98.32%)	
		Width	44.2266	44.8770	(101.47%)	44.7700	45.2866	(101.15%)	
	Egg 7	Thickness	1.3008	1.1382	(87.50%)	0.3400	0.2664	(78.35%)	
		Height	57.8848	57.2344	(98.88%)	57.8200	58.8440	(101.77%)	
		Width	44.2266	43.5762	(98.53%)	43.6300	44.1922	(101.29%)	
	Egg 8	Thickness	0.9756	0.9756	(100.00%)	0.2900	0.3108	(107.17%)	
		Height	57.2344	55.2832	(96.59%)	57.5300	58.1743	(101.12%)	
		Width	44.2266	44.2266	(100.00%)	44.2800	44.2006	(99.82%)	
	Egg 9	Thickness	1.1382	1.3008	(114.29%)	0.3600	0.2259	(62.75%)	
		Height	59.1855	58.5351	(98.90%)	59.3300	59.3228	(99.98%)	
		Width	46.1777	46.8281	(101.41%)	46.5800	44.6234	(95.80%)	
	Egg 10	Thickness	1.1382	1.3008	(87.50%)	0.3300	0.2882	(87.33%)	
		Height	56.5840	54.6328	(96.55%)	55.7600	56.4656	(101.27%)	
		Width	42.9258	43.5762	(101.52%)	44.0800	43.3312	(98.30%)	
	Egg 11	Thickness	1.4634	0.9756	(66.67%)	0.3600	0.2100	(58.33%)	
		Height	57.8848	56.5840	(97.75%)	58.2000	58.7781	(100.99%)	
		Width	43.5762	42.9258	(98.51%)	43.5300	44.3173	(101.81%)	
	Mean ± sd	Thickness			(82.61% ± 17.11)			(86.29% ± 21.40)	
		Height			(98.88% ± 1.38)			(100.21% ± 1.32)	
		Width			(100.55% ± 1.72)			(99.65% ± 1.82)	

## Data Availability

The code used to obtain these results has been made available in a github repository at reference [37].
